# Supplementary material for: Content-rich biological network constructed by mining PubMed abstracts
Source: BMC Bioinformatics. 2004 Oct 8;5:147. doi: 10.1186/1471-2105-5-147 (PMC528731; doi:10.1186/1471-2105-5-147)
Supplement: Additional File 2 — The original results of the above study (non-essential files are deleted to keep the file size under the limit set by BMC bioinformatics). [file 1471-2105-5-147-S2.bz2 › chilibotAdditionalFile2/dip10/391ID10498602E9873/html/NRP1_SEMA3A.html]

 


 **NRP1** and **SEMA3A** 
  
Found 69 abstracts in PubMed, retrieved 10.  
 

 What does Google say? 
 PDF only 
| .edu only 

---

**Interactive relationship** (e.g. stimulation, inhibition, etc)

**Neutral relationship**- COS 7 cells coexpressing plexin A4 with neuropilin 1  [ **NRP1** ]  were induced to contract by  **Sema3A** , a member of the class 3 semaphorin.  Ref: 12591607 Mech Dev, 2003
- Semaphorin 3A  [ **SEMA3A** ]   **Sema3A**  repels growing olfactory axons that express neuropilin 1  [ **NRP1** ]  NP 1, a receptor for  **Sema3A** .  Ref: 12598627 J Neurosci, 2003
- Expression of sema3a1 in somites and neuropilin 1  [ **NRP1** ] , which encodes for a component of the  **Sema3a**  receptor, in angioblasts suggested that Sema3a1 regulates the pathway of the dorsally migrating angioblasts.  Ref: 12783793 Development< MedlineTA>Development, 2003
- Neuropilin 1  [ **NRP1** ]  Npn 1 is a receptor that binds multiple ligands from structurally distinct families, including secreted semaphorins Sema  [ **SEMA3A** ]  and vascular endothelial growth factors VEGF.  Ref: 12852851 Dev Cell, 2003
- VEGF165 and  **Sema3A**  are competitive inhibitors for  **NRP1**  mediated functions in EC and neurons.  Ref: 12613541 Adv Exp Med Biol, 2002

**Non-interactive relationship** (e.g. studied together, co-existance, homology, etc.)

- Chemorepulsion from the ventral spinal cord does not depend on  **Sema3A**  neuropilin 1  [ **NRP1** ]  but requires axonin 1 SC2 to repel DRG axons.  Ref: 12591248 Dev Biol, 2003
- The dermamyotome derived chemorepulsion is exclusively dependent on  **Sema3A**  neuropilin 1  [ **NRP1** ] .  Ref: 12591248 Dev Biol, 2003
- After the initial discovery of neuropilin 1  [ **NRP1** ]  as an epitope on axons recognized by a monoclonal antibody, neuropilins were rediscovered in the search for receptors mediating the repulsive actions of class 3 Semaphorins, notably  **Sema3A** .  Ref: 12613540 Adv Exp Med Biol, 2002
- Using coculture assays composed of tissues derived from the chick embryo or mutant mice treated with function blocking antibodies and phosphatidylinositol specific phospholipase C, we report here that the chemorepellent semaphorin 3A  [ **SEMA3A** ]   **Sema3A**  and its receptor neuropilin 1  [ **NRP1** ]  are required for mediating the dermamyotome and notochord derived, but not the ventral spinal cord derived, chemorepulsive signal for DRG axons.  Ref: 12591248 Dev Biol, 2003
- Second, L1 and  **NRP1** , the neuropilin responsible for  **Sema3A**  binding, associate through their extracellular domains, forming a cell surface heterocomplex.  Ref: 12613546 Adv Exp Med Biol, 2002
- Neuropilin 1  [ **NRP1** ]  and plexin As form receptor complexes for  **Sema3A**  and plexin As are thought to initiate the intracellular signaling cascade.  Ref: 12559962 Biochem Biophys Res Commun, 2003
